# Legal perspectives on liability for medical negligence and malpractices in Nigeria

**DOI:** 10.11604/pamj.2020.35.44.16651

**Published:** 2020-02-17

**Authors:** Oludamilola Adebola Adejumo, Oluseyi Ademola Adejumo

**Affiliations:** 1 Faculty of Law, Obafemi Awolowo University, Ile-Ife, Nigeria; 2 Department of Internal Medicine, Faculty of Clinical Sciences, University of Medical Sciences, Ondo City, Nigeria

**Keywords:** Legal, medical, liabilities, negligence, malpractice

## Abstract

Medical practice usually involves different activities which if not professionally handled, may give rise to liabilities on the part of the medical practitioner. These liabilities may arise in tortious claims and in some other cases, may go beyond the realm of civil liabilities to criminal liabilities. This review focuses on liabilities that amount to negligence both under the civil and criminal laws in Nigeria, other instances of malpractices which may not amount to negligence but may suffice to give rise to a successful cause of action in other branches of substantive law including claims for breach of fundamental human rights; contract; and fiduciary relationship. The review concludes by emphasizing the need for caution and the need to ensure that justice is seen to be done not only to the victims but also to the medical practitioners who deserve all legal protection in the exercise of their professional duties.

## Introduction

Medical negligence is hinged on the tortious principle of negligence as propounded by Lord Atkin in the 1932 case of Donoghue V. Stevenson [1]. Medical negligence constitutes an act or omission by a medical practitioner which falls below the accepted standard of care resulting to injury or death of the patient [2]. The case established a general duty to take reasonable care to avoid foreseeable injury to another. Therefore, to establish a case in negligence, it must be shown that a duty of care was owed; there had been a breach of that duty; and that damage or injury was suffered as a direct result of a breach of the duty owed. In medical negligence and going by the definition above, medical practitioners who undertake the care and treatment of patients owe a duty of care to such patients. A duty of care is necessarily implied when a patient is registered and being treated in a hospital. The view has been expressed that, care of medical practitioners ought not to be limited only to the patients under their direct management but to be extended to any patient whom they come across in their professional environment and as such, a medical practitioner owes the duty to care for every patient found within the hospital premises whether or not he is on the management team of such patient [3]. This view appears extreme. However, the fact that this may minimize incidents of direct or vicarious liability by a hospital as an entity for negligence may make this view desirable to some extent.

In medical practice, the standard of care required is usually contained in the rules of professional ethics for medical practitioners in different regions. In Nigeria, the standard of care is set by the Medical and Dental Council of Nigeria. Other medical bodies including the Nigerian Medical Association, the Medical and Dental Consultants Association of Nigeria also have principles of ethics guiding their members with disciplinary measures in place to ensure compliance. This review focused on discussion of issues in negligence, medical errors and malpractices while highlighting other legal remedies available to a victim where negligence cannot be proved. The first part of the paper discusses the various remedies available in cases of medical errors and malpractices. The second part highlights the various issues in establishing and proving the standard of care required of medical practitioners and the breach of such duty. This part also discusses issues of causation, discharge against medical advice, vicarious liability and *res ipsa loquitor.* The third part of the paper goes beyond the limit of civil liabilities to discuss the criminal aspects of negligence. The fourth part highlights the findings of the review and the fifth part concludes with recommendations.

## Methods

This review was conducted using both peered reviewed and grey literature focusing on research evidences derived from the fields of Law and Medicine especially in Nigeria. An electronic literature search was conducted in the following databases: Google, Google Scholar, PubMed, and Jstor stable. Key words that correspond to the thematic objectives of the review were used in the search including medical negligence, standard of care, duty of care, criminal negligence, doctor-patient relationship and health related rights. Eligible articles were included for review only when abstracts contained explicit information about the issues of interest. Full text of the relevant articles and literature were then accessed and read. Also, relevant laws within the Nigerian legal system were reviewed and provisions relevant to the review were highlighted in the review. The review also included detailed analysis of existing judicial decisions and case law relating to the issue of interest.

## Current status of knowledge

**Medical negligence and medical errors/malpractices:** medical errors occur when a medical practitioner chooses an inappropriate method of care or improperly executes an appropriate method of care [2]. A medical error is a commission or an omission with potentially negative consequences to the patient that would have been judged wrong by skilled and knowledgeable peers at the time it occurred, independent of whether there were any negative consequences [4]. Going by the above, there seems to be a very thin line between acts that constitute medical negligence and medical errors or malpractice. Acts that constitute medical errors may or may not give rise to a claim in medical negligence. Under the extant principles of negligence, not all medical errors and malpractices will qualify as an act of negligence. For instance, a medical error may not have given rise to any injury or damages and thus, a claim of negligence hinged solely on such an act is unlikely to succeed. Such an act may however give rise to a disciplinary action against such medical practitioner by professional bodies such as the Medical and Dental Practitioners’ Disciplinary Committee hinged on a breach of medical ethics. As such, breach of medical rules and ethics may not suffice to necessarily give rise to a claim in negligence. It is thus important to discuss the alternative options available to a person who is unable to successfully establish negligence but who has been a victim of medical wrong or malpractice.

**The contractual angle:** apart from the above, patients who have suffered some form of damage or injury in the course of treatment may bring an action for breach of contract. This may be a viable option especially in cases where negligence cannot be proved. Inherent in doctor-patient relationships that is largely contractual is an implied term that the doctor will exercise reasonable skill and care in the treatment of patients [5]. As such, the law will imply the existence of a contract in cases where a patient submits to treatment by a medical practitioner [6]. The rationale for this as seen in most breach of contract cases is that the defendant is made to put the patient in the position he would have been if treatment was properly performed. The claim for damages will also lie where the breach of the medical practitioners’ warranty has caused the patient to incur some extra costs. To succeed in an action for breach of contract unlike in negligence cases, it suffices for the patient to prove the existence of a doctor-patient relationship; breach of the implied /express term of the contract- to treat; and injury arising from or in the course of treatment.

**Fiduciary relationship:** under the rules of equity, a claim may also be hinged on the recognition of a doctor-patient relationship as one which imposes a fiduciary duty on the medical practitioner. A fiduciary duty to protect the patients’ interest may be imposed on the medical practitioner in favour of the patient. This was successfully done in Norbery V. Wynrib [7] where the court upheld this view to uphold and defend the patient’s fundamental and personal interest [5]. There are also cases where the patient suffers damages or injury but has no valid claim against the medical practitioner. This will arise where the patient has given informed consent or where the medical practitioner acted based on compulsion to save the life of the patient. An apt example will be the removal of a patient’s uterus which refuses to contract during a caesarean section operation. The medical practitioner’s action is unlikely to amount to negligence or breach of his fiduciary duty especially in circumstances where his actions were in good faith and in the best interest of the patient.

**The human rights angle:** liability for medical error or malpractice may also validly arise as a breach of a patient’s fundamental human right. The relevant basic fundamental rights of a patient must be borne in mind and safely guarded, in the course of their treatment by medical practitioners. The patient’s autonomy should also not be disregarded by attending physicians. The right of the patient to make final and conclusive decision about his medical care is well recognized under the principle of patient’s autonomy and also, well enshrined in the fundamental human rights of persons [8, 9, 10]. The right to personal liberty and self-determination may also be implied in some medical cases to buttress autonomy. The use of a right-based approach to deal with issues in medical practice is not to “play the blame game” or to punish erring individuals but primarily to form a basis for practical accountability on the part of government and health care providers in the provision of health care services to citizens. This will result in safe, functional and effective health care systems [11]. The right to health has been widely interpreted to include the right to freely make decisions on issues pertaining to one’s health and to have access to information on one’s health issues and available treatment options [12]. Failure to provide information on all available treatment options may thus give rise to liability for negligence and breach of the patients’ right to health. A duty is owed by the medical practitioner to inform a patient for instance of the new knowledge of risks of products [13]. The 2014 National Health Act in Nigeria contains provisions that emphasize the right of a patient to be informed of his health status; treatment options available; the benefits, risks, costs and consequences of such options; and the right to refuse treatment after detailed explanation of implication of refusal by the medical practitioner to the patient [14].

The right to privacy has been held by the courts to include the right of a mature adult to refuse treatment that may prolong his life even though such refusal may seem unwise, foolish or ridiculous to others [15]. In a decided Nigerian case, the court noted the refusal of courts in most cases to override the patients’ decision and thus, emphasized the supremacy of patients’ autonomy [16]. In McGlinchey V. UK [17] the standard of care was held to be in breach of Article 3 of the European Convention on Human Rights which guarantees the right of every person to be free from cruel, inhuman and degrading treatment. Failure to prevent or protect patients from causing harm to themselves may also amount to some form of cruel treatment. The question whether systems and standards are in place suitable enough to protect patient will be material in determining liability. For instance, the question of whether patient was kept under routine surveillance would be material to determine liability where a patient commits suicide [18]. Going by the above discussions, it is clear that the victims of medical errors or malpractices not necessarily amounting to negligence may resort to any of the above options to seek appropriate legal remedy.

**Standard of care and breach of duty of care:** usually, the standard used is that of the ‘reasonable man’-that is, that of an ordinary person placed in the same circumstances. In terms of medical negligence however, the focus is on the standard of professional duty expected of a comparable medical practitioner. The argument has been raised that the standard expected of a house officer or a young medical officer/resident should not be the same standard expected of a Consultant. In a decided case, the standard expected of a learner for instance, was held to be different from that required of a professional driver [19]. As such, the Consultant ought to be a specialist in a chosen field and hence, the degree of care expected of him should thus, be higher than that of a non-specialist and this should not be overlooked in determining liability. An exception may however arise in cases where a junior doctor is undertaking provision of specialist services; the standard that will be required in such circumstances may be that of a specialist whilst also not overlooking the liability of the hospital to employ the services of qualified specialists to provide specialist care and needed supervision, when necessary [20]. In any event, the court will be in the position to consider the peculiar circumstances of each case. Issues of necessary supervision; considerations of referrals etcetera will be considered to determine liability. A junior doctor’s obedience to manifestly wrong instruction given by a superior may be construed against the junior doctor as negligence. This is obviously because the junior doctor is deemed to have been in a position to reasonably foresee that harm or injury will be caused. The operative word here is ‘manifestly’ wrong. Ordinarily, where specific instructions are given to a junior doctor by his Consultant, he ought not to depart from such instructions and departure must be reasonably justifiable [21]. In cases where a junior doctor chooses to depart from his superior’s instruction, without reasonable justification, he will be solely held liable if his actions resulted in negligence. His superior may only be liable vicariously where negligence can be established on his part as a result of not giving adequate supervision to his junior. As expected in civil matters, the standard of proof required is the proof on the balance of probabilities which requires the judge to weigh the totality of evidence adduced by both parties to ascertain in whose favour the scale of justice tilts. In emergencies, good medical practice requires a medical practitioner to offer assistance taking into account his safety, competence and availability of other options opened to the patient. However, judicial backing has been given to the fact that errors of judgment are more excusable in emergencies than in other cases [22].

Mistake in diagnosis will also not amount to negligence if the required standard of care has been duly observed. In cases where there is some form of doubt on the part of the medical practitioner as to specific diagnosis to make, such a person ought to make a referral to a specialist [20], failure to do so may however amount to negligence. The standard of care required from alternative medical practitioners appears to be lenient especially where the act is not such that will give rise to liability for criminal negligence. In Shakoor V. Situ [23], the court held that an alternative medical practitioner could not be judged by standard of an orthodox medical practitioner. The rationale for this is that the alternative medical practitioner has not by his practice held out himself as professing the art of medicine in the orthodox sense and as such, the standard required of him is that which is prevalent in the art of alternative medical practice. This, as will be shown below, in our discussions on criminal negligence will appear not to be the case in Nigeria especially as regards actions which amount to criminal negligence. A breach of duty is established where a medical practitioner’s actions has failed to meet an appropriate professional standard. The determination of appropriate standard is not fixed and as noted above, it may be subject to certain facts. In the case of Bolam V. Friern Hospital Management Committee [24], the court was of the view that it suffices, if a doctor acted in accordance with a practice that was considered acceptable by a responsible body of doctors [25]. The burden is on the claimant to show that no reasonable doctor acting in the same circumstances would have acted in the way the defendant acted. The fact that the culpability of a medical practitioner is largely dependent on the expert evidence of a colleague has been largely criticised on the grounds that the approach seems to be in favour of the medical profession over and above the patient and hence, support from colleagues arguably makes it easy to escape liability for negligence. While this seems like a possibility, the fact that judges have the prerogative to determine the weight to attach to evidence adduced in a suit cannot be overlooked. In essence, where evidence given appears tainted, the judge has a responsibility to disregard such evidence. This was evident in the court’s decision in Hucks V Cole [26] where it rightly held that ‘the court must be vigilant to see whether the reasons given for putting a patient at risk are valid or whether they stem from a residual adherence to out of date ideas’ [6]. In the same vein, the court in Bolitho V. City and Hackney [27] held the view that negligence can be successfully proved even in cases where medical opinion suggests otherwise. The court emphasized the need for the judge to consider evidence adduced and decide whether the action unnecessarily puts patients at risk. In establishing whether a breach has occurred, the courts can also rely on written guidelines and rules of medical ethics to ascertain standard practices.

**Issues in causation:** the fact that the Claimant’s injury was caused by the medical practitioner is crucial to establish negligence. Not only should the injury be caused by the defendant, the injury must be a direct and not a remote consequence of the defendant’s action. Hence, Lord Denning in M V. London Borough of Newham [28] rightly noted that causation is a question of fact and not law. This is especially relevant in circumstances where the Plaintiff would have died or inevitably sustained injury irrespective of the defendants’ negligence. Causation cannot be based on assumptions especially in cases of medical negligence and hence, must be proved or at the minimum, show that the claimant’s injury was caused substantially by the defendant’s actions [29]. In Barnett V. Chelsea and Kessington Hospital Management Committee [30] where a medical practitioner failed to attend to some patients who presented at his clinic, resulting in the death of one of the patients before morning, the court held that the medical practitioner did not cause the death of the said patient. This was particularly because there was no known cure for the patient’s ailment and the patient would in any event had died even if he was attended to. The issue of causation will also be required to be settled in cases where there are alternative possible causes of death or injury. Proof that the medical practitioner’s negligence caused the injury or death cannot be dispensed with in such cases [21]. The medical practitioner’s ability to reasonably foresee damage or injury is also crucial in proving causation and establishing negligence.

**Res Ipsa Loquitor:** the plaintiff in civil case of negligence can make a plea of *res ipsa loquitor-* meaning ‘the fact speaks for itself’. This is an exception to the requirement of proof in certain cases. The plea of *res ipsa* is to the effect that the plaintiff’s situation is deemed to indicate that it was clearly a consequence of the defendant’s negligence. As such, the burden shifts to the defendant to rebut the presumption of negligence against him by showing that the plaintiff’s situation could have been or was caused by other factors. The court is usually reluctant to extend *res ipsa loquitor* doctrine to cases of medical negligence. This is particularly because of the nature of the human system and medical practice. It may be easier to make such plea in cases where things are purely ‘physical’ and can be glaring enough to see. However, by the nature of medical cases, it is not usually very easy to conclusively plea *res ipsa loquitor.* In O’Malley-Williams V. Board of Governors of National Hospital for Nervous Diseases [31], the plea of *res ipsa* failed because the injury being complained of was a well-recognised consequence of the procedure that was carried out. Be that as it may, the doctrine of *res ipsa* may suffice in some exceptional medical negligence cases, to shift the burden of proof from the victim, to the medical practitioner.

**Hospital managements’ liability:** apart from the liability of medical practitioners in their individual capacities, a hospital may also be liable for negligence. This is especially because hospitals are no longer being regarded solely as ‘venues for treatment’ but as ‘providers of treatment’ [32]. This development has given rise to the liability of hospitals either directly or vicariously for acts of negligence. Direct liability for negligence will arise where a hospital has failed to provide an environment and facilities that will facilitate safe treatment of patients. For example, this will arise where equipment which are expected to be available are not available or are not functional leading to harm, injury or death of patients. Examples include: a non-functional ambulance, unhygienic conditions, non-maintenance of medical records, and transmission of infections amongst others [31, 33, 34]. Vicarious liability on the other hand will arise where the hospital is being held liable for acts, omissions and failure of its staff, in the discharge of their responsibilities in the hospital. This view was well expounded by Lord Denning in the 1951 case of Cassidy V. Minister of Health [35]. A senior medical practitioner may also be held vicariously liable for the actions or omissions of a junior or any member of the medical team that he leads or who is under his supervision and control [5].

**Discharge against medical advice:** the basis for legally administering treatment on a patient is hinged on the fact that the patient whether expressly or impliedly gives his consent. In law, treatment is not to be administered without consent and it is not sufficient excuse that it was done for the benefit of the patient [5]. Discharge against medical advice (DAMA) is a recognized phenomenon in hospitals with potential medico-legal implications on the hospital authority and medical staff [36, 37]. Both the Professionalism Charter and the law recognize that patients are mature individuals who have the right to take a DAMA, for which the attending physician may incur liabilities where he opposes without reasonable justification. In the exercise of such rights however, medical staff must be wary of avoiding deficiencies and must put in place proper procedures and documentation of cases where the patients insist on DAMA. Law suits related to discharges seem more common among those discharged against medical advice [38]. Well-executed DAMA forms have been found to protect physicians against litigation and indeed, will be a useful and compelling piece of evidence to help establish a defence for the physician from any liability in any civil suit which may be instituted against him [39]. Prescribed procedure is that DAMA should be administered by the attending physician. Indeed, if possible, because of the sensitive nature of the process, the most senior doctor should administer the document [38]. In some cases, where the patient or the family feel the closeness and empathy of the experienced physician, the decision to DAMA may be reversed. The physician is expected to assess the DAMA form for adequacy and proper filling [40] and failure to do so may be fatal where defence in an action on negligence is hinged on DAMA. In situations where the patient refuses to sign the DAMA form, the content should be read out aloud and patient’s refusal to sign documented; the fact that the patient was made aware of the risks of leaving should also be documented [38]. Inability to properly administer the DAMA form as part of the discharge process is equivalent to an act of negligence with legal consequences [38]. Indeed, the need for the patient to be well informed prior to signing the form cannot be over-emphasized and thus, the signing of the DAMA form should only be a confirmation that a detailed conversation which had helped the patient come to the decision to seek DAMA has taken place between the patient and the physician [41]. Until that is done, the patient cannot be said to have enjoyed his full autonomy and the medical personnel may be culpable in a law court for infringement of the patient’s fundamental human rights and more specifically, liability for negligence.

**Criminal negligence:** apart from civil liabilities which have been our focus, so far, a medical practitioners’ action may also result in commission of a crime giving rise to criminal liability. Liability may arise for instance for criminal assault or for causing grievous bodily harm. Hence, where in the course of treatment, and due to some form of negligence on the part of the medical practitioner, a patient suffers some gross or extreme harm or death, showing disregard for life and safety, liability will arise under criminal negligence [42]. This view was given expression by the Privy Council in the Nigerian case of R. V. Akerele (supra) where the court held that the degree of negligence required in criminal cases must go beyond that for civil liability and it must be shown that there has been ‘such disregard for life and safety of others’ to amount to manslaughter. This is *in tandem* with the rule of evidence relating to standard of ‘proof beyond reasonable doubt’ for criminal cases. The view has been expressed that liability for criminal negligence is limited to prosecution for manslaughter [21]. In Nigeria however, it appears based on the provision of Section 343 of the Nigerian Criminal code [43], that liability will arise in criminal negligence for acts other than manslaughter. Section 343 is to the effect that any person who gives medicine or medical or surgical treatment in a rash or negligent manner as to endanger life or likely to cause harm to a person shall be guilty of a misdemeanour. As such, under Nigerian criminal law system, liability will arise even where life has not been lost but endangered, in the course of treatment. Also, Section 303 of the Nigerian Criminal Code requires that persons who undertake to administer surgical or medical treatment should possess reasonable skill and use reasonable care in acting except in cases of necessity. This can on the face of it be interpreted to accommodate or recognise persons other than qualified medical practitioners for instance, quacks, to carry out surgical and medical treatment provided they use reasonable skill and care. A second look at the provision will however reveal that the requirement for possession of reasonable skill and use of reasonable care is to be read conjunctively and not in the alternative. Thus, the view has been expressed by some that the test for judging responsibility is not a person’s qualification or skill but a person’s conduct considered negligent [5]. Thus, the decision reached by the court in the case of R V. Lawanta [44] where the defendant was acquitted on a charge of manslaughter because the court found that although unqualified, he exhibited the proper degree of skill by sterilising equipment used is considered questionable, in view of the express provision of Section 303. Sterilising of equipment does not suffice to establish requisite skill in handling treatment involving human life. The fact that the accused was not qualified immediately suggests that he could not have possessed the reasonable skill required under section 303.

It must be noted however that skills do not only involve possession of qualifications; it may be a product of years of experience which ought not to be assumed or dispensed with or substituted with use of reasonable care. A locally trained mid-wife who has taken multiple deliveries may be able to exhibit reasonable skill in taking delivery. However, it is our submission that the issue of possession of reasonable skill especially for informally trained persons should be one to be proved sufficiently and undoubtedly by careful consideration of the facts and circumstances of each case, before a decision is reached. This will be in recognition of the sanctity of human life and the need to protect same. Happily, the court in R V. Ozegbe [45] was stricter in construing the provision of section 303 and the defendant was convicted for manslaughter as he had no proper knowledge of the surgery which he carried out. The view has been expressed that a clear distinction should be made between cases of recklessness and cases of criminal negligence arising from sheer ignorance or incompetence. Mason and Laurie [46] for instance have suggested that cases of sheer ignorance or incompetence resulting in commission of crime may not in all cases be strongly deserving of punishment and that professional sanctions may suffice in such cases. This argument is hinged on the fact that there will be no evidence of wrong doing on the part of the medical practitioner. In New Zealand, the Crimes Act has been amended to require substantial departure from normally expected levels of care to establish liability for manslaughter [47]. In the Nigerian context, this view is however not yet tested as existing cases showed that the court has ruled otherwise [46]. This may be particularly because the view appears to be inconsistent with and a clear departure from the provisions of sections 303 and 343 of the Nigerian Criminal Code.

This study finds that not all wrongs committed by a medical practitioner can successfully give rise to a claim in negligence. This is particularly because the standard of proof required to establish the tripartite requirements of negligence is a burdensome one which the victim must discharge. More so, it is not in all cases that a person may be able to prove that he suffered actual damages even though some wrong had been done to him in the course of receiving treatment. This study particularly notes that the reasonable man’s test appears to be unduly protective of the medical practitioner and this may or may not be to the detriment of the patient, depending on the circumstances of the case. This study finds that improper execution of DAMA suffices to give rise to a claim in negligence and that the doctrine of *res ipsa* may not suffice in medical negligence cases to shift the burden of proof from the victim, to the medical practitioner. This is because of the complex nature of medical practice that goes beyond what can be physically seen and assessed. Furthermore, our study finds that claims for medical errors and malpractices not amounting to negligence in law, can be successfully proved by reliance on the principles of fundamental human rights, contract and equity. The final finding of the study is the fact that the criminal laws relating to medical negligence in Nigeria makes no distinction between cases of recklessness and cases of criminal negligence arising from sheer ignorance or incompetence. As such, same punishment will apply to someone found to be reckless and one who was simply ignorant or incompetent. Thus, the demands under Nigerian laws are stricter than some other jurisdictions.

## Conclusion

It is almost clear that medical practice will sometimes give rise to situations where patients suffer some harm or injury in the course of treatment by the medical practitioners. This harm or injury may be caused either by commission or omission of some actions done by or failed to be done by the medical practitioner. For liability to arise in negligence, it is clear that the three core elements of negligence must be established and proved. However, failure to successfully establish a claim in negligence does not exonerate a medical practitioner from liability for malpractices or errors under other branches of the law. As seen above, the fact that negligence cannot be proved will not leave a patient without other legal remedies, as may be deemed appropriate. Notwithstanding the above, the law protects the medical practitioners to the extent that liability for criminal negligence must be proved beyond reasonable doubt and also, in civil cases, the ‘reasonable man’s test’ is deemed to be largely protective of the medical practitioner over and above the patient to a large extent. In all, the need to relieve the victim who has to incur litigation expenses and suffer the rigours of litigation must be emphasized. This can be done, for instance, in Nigeria by the adopting of the ‘no fault’ compensation scheme as is being done in New Zealand. This scheme relieves patients of the need to establish fault on the part of the medical practitioner once injury is suffered. The UK NHR Redress Act 2006 which introduces the redress scheme as an alternative to litigation in less severe cases of negligence are also recommended for adoption in other jurisdictions to relieve patients of the rigours of litigation in certain cases where negligence is apparent. There is also a need to review the criminal laws on negligence resulting to manslaughter to distinguish between cases of recklessness and cases of criminal negligence arising from sheer ignorance or incompetence.

### What is known about this topic

- Existing literature focuses majorly on medical negligence which is a tortious wrong for which medical practitioners may be liable where a patient has suffered some harm or injury in the course of treatment;
- The task of successfully establishing negligence is usually a burdensome one which requires the patient to prove the three elements of negligence-existence of a duty of care, breach of duty and damages arising from the breach;
- Apart from liability for civil claims, liability may also arise for criminal negligence where the acts of the medical practitioner results in the commission of a crime under criminal laws.

### What this study adds

- This study provides alternative causes of action through which a claim may be brought successfully against a medical practitioner for medical malpractices and errors;
- The study establishes that there are no distinctions between cases of recklessness and criminal negligence arising from sheer ignorance or incompetence under the Nigerian legal framework;
- The study provides alternative compensation and redress scheme which will relief patients from the hurdles of litigation in cases of medical malpractices and error.
